# Supplementary material for: Profiling human breast epithelial cells using single cell RNA sequencing identifies cell diversity
Source: Nat Commun. 2018 May 23;9:2028. doi: 10.1038/s41467-018-04334-1 (PMC5966421; doi:10.1038/s41467-018-04334-1)
Supplement: Supplementary file 1 — Supplementary Information [file 41467_2018_4334_MOESM1_ESM.pdf]

Supplementary Figure 1

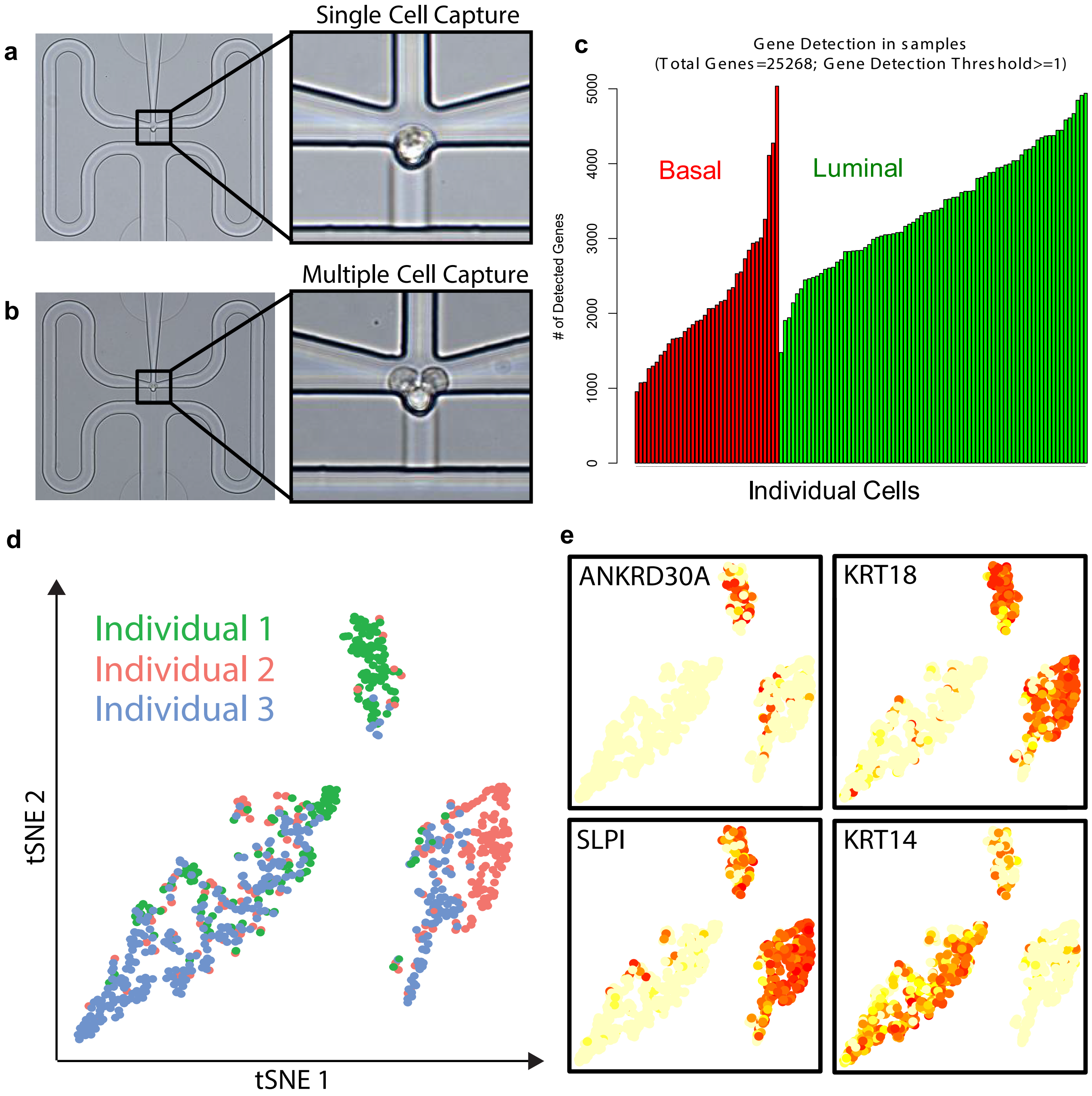

Supplementary Figure 1. Technical information and supportive data on microfluidics-enabled scRNAseq. (a-b) All 96 capture sites were imaged using the Keyence BZ-X700 microscope to confirm single cell capture **(a)**, and to exclude capture sites that contained doublets or multiplets **(b)**. **(c)** Number of genes detected per cell were distributed in comparable manner between basal (red) and luminal cells (green). **(d)** tSNE projection of data generated on microfluidics-enabled scRNAseq data, with cells colored by the individual sample source. **(e)** Feature plots showing the scaled expression of ANKRD30A marking cell type L2, KRT18 marking both luminal cell types, SLPI marking cell type L1 with greater specificity, and KRT14 showing the highest expression in basal cells.

Supplementary Figure 2

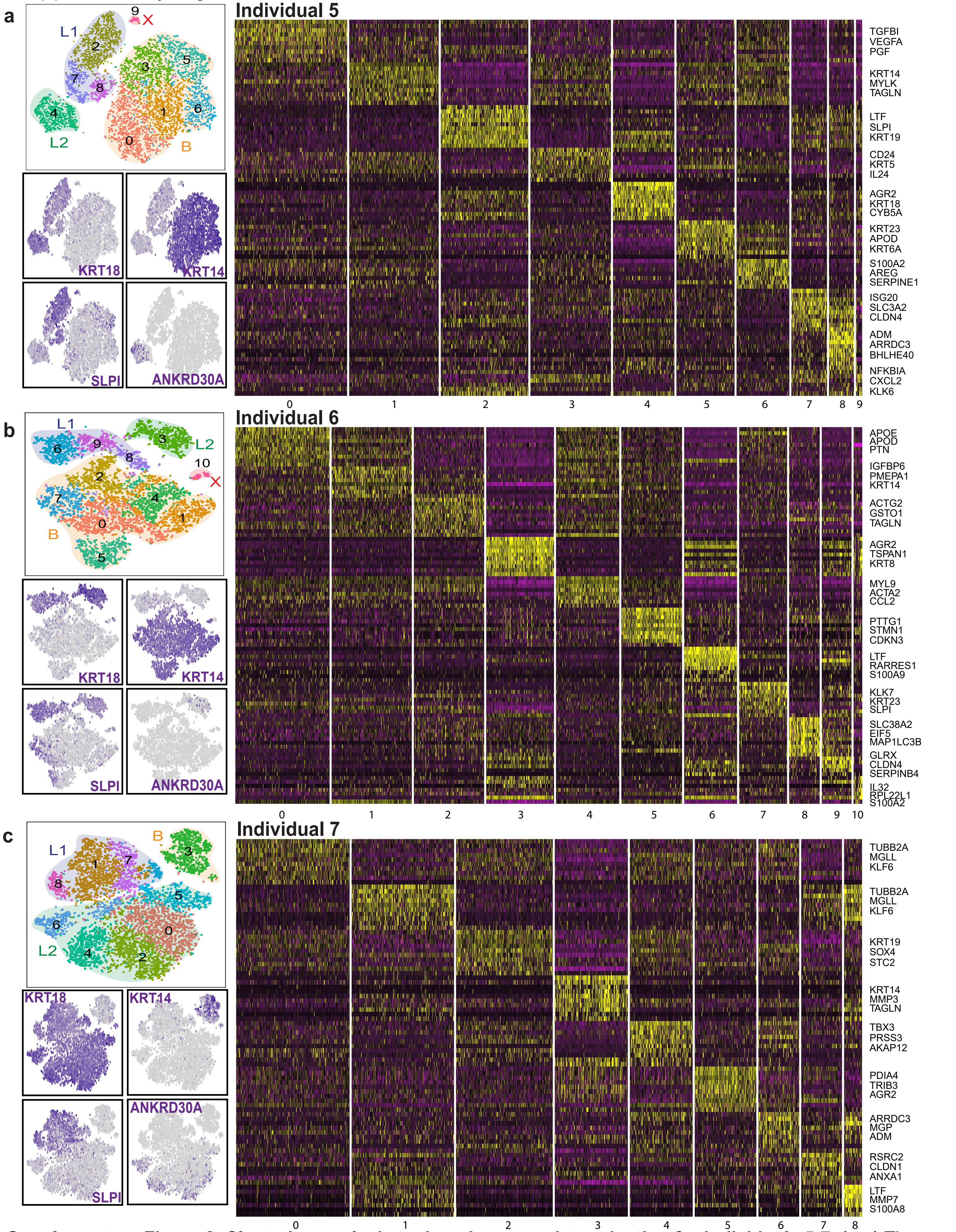

Supplementary Figure 2. Clustering analysis and marker gene determination for individuals 5-7. (a-c) The individual data matrices for Individual 5 **(a)**, 6 **(b)**, and 7 **(c)** were analyzed using Seurat and their initial cluster determinations are displayed using tSNE projection. Feature plots of characteristic markers of highlighting the three main cell types Basal, L1 and L2 are shown. Additional less frequent non-epithelial populations were detected in some individuals and were designated unclassified (X). Heatmaps showing the top 10 marker genes of each cluster is displayed highlighting selected marker genes for each cluster.

Supplementary Figure 3

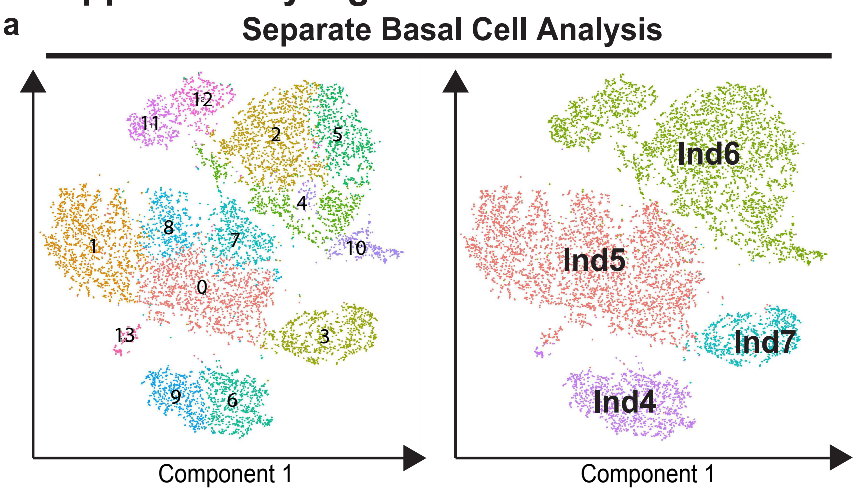

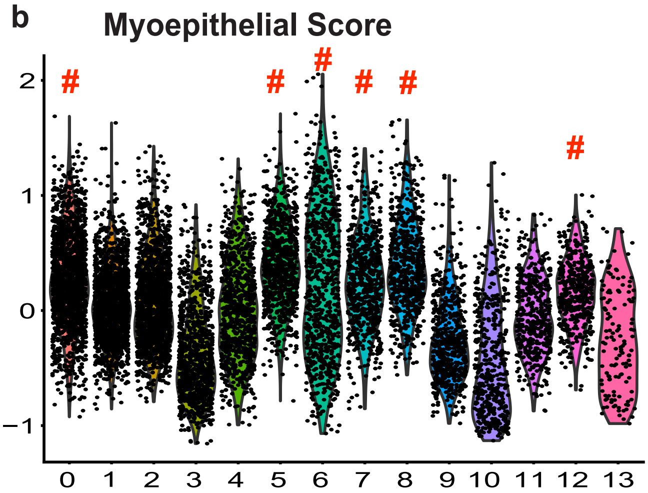

c

Clusters from Individual Analysis

Ind4	Ind5	Ind6	Ind7	Cell State
**3**	0,1,6	0,1,4,5	3	
**4**	3,5	2,7	3	Basal
**5**	-	8	3	
**0**	2	6	7,8	Luminal1.1 (L1.1)
**1**	2	6	7,8	
**2**	7,8	9	1	Luminal1.2 (L1.2)
**6**	4	3	0,2,4,5	Luminal2 (L2)
**7**	4	3	6	
**8,9,10**	9	10	-	Unclassified (X)

Clusters from separate Basal Analysis

Clusters	Cell State
1,2,3,4,9,10,11,13	Basal (B)
0,5,6,7,8,12	Myoepithelial (Myo)

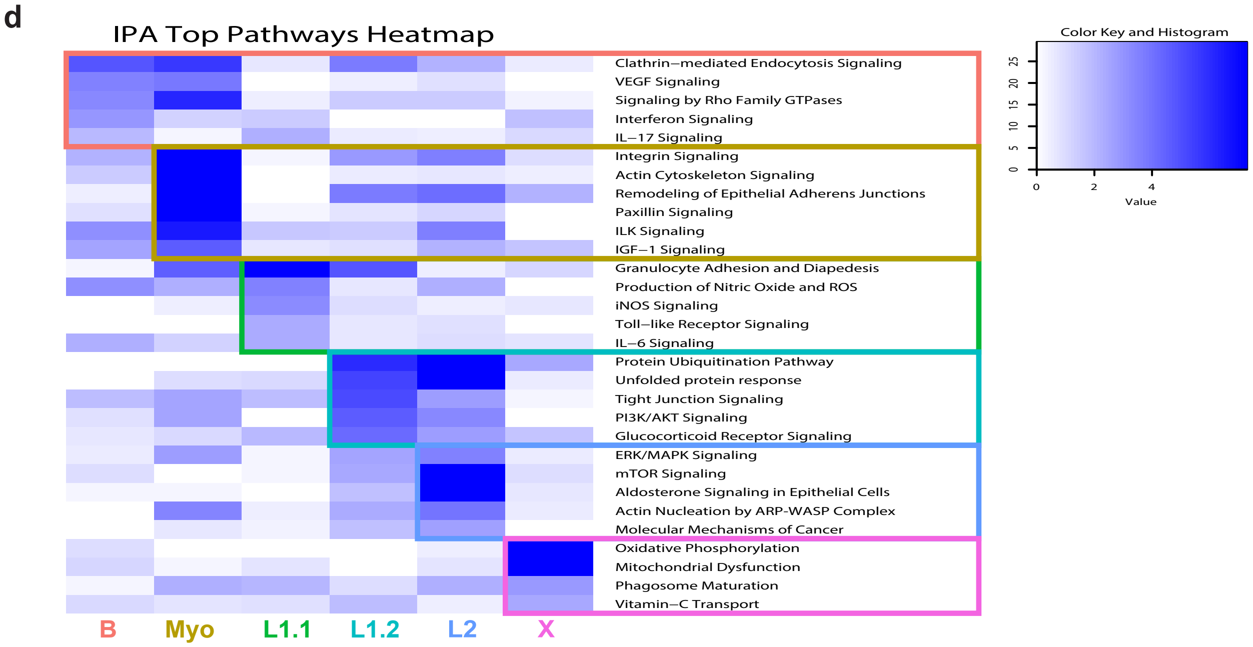

Supplementary Figure 3. Combined basal cell only analysis and ingenuity pathway analysis (IPA). (a) Basal cell clusters (KRT14+) from all four droplet-enabled scRNAseq datasets were combined and analyzed using Seurat. tSNE projections and of cells belonging to the basal cell lineage across all individuals in a combined analysis, colored by cluster determination and individual library source. **(b)** Violin plots showing the gene scoring results for a curated Myoepithelial gene signature (see Supplementary Data 2) was used to stratify regular basal cells from myoepithelial clusters(marked by #). **(c)** Summary of individual cluster matches and final cluster assignments as indicated in “Cell State” column. Basal cell populations were separately analyzed and then scored using a myoepithelial signature gene list, resulting in the final cell state determinations of Basal (B), Myoepithelial (Myo), Luminal1.1 (L1.1), Luminal1.2 (L1.2), Luminal2 (L2), and Unclassified (X). **(d)** Heatmap showing log-scaled p-value of enrichment for IPA annotated pathways, processed via comparison of IPA expression enrichment analysis on marker genes for each cluster.

Supplementary Figure 4

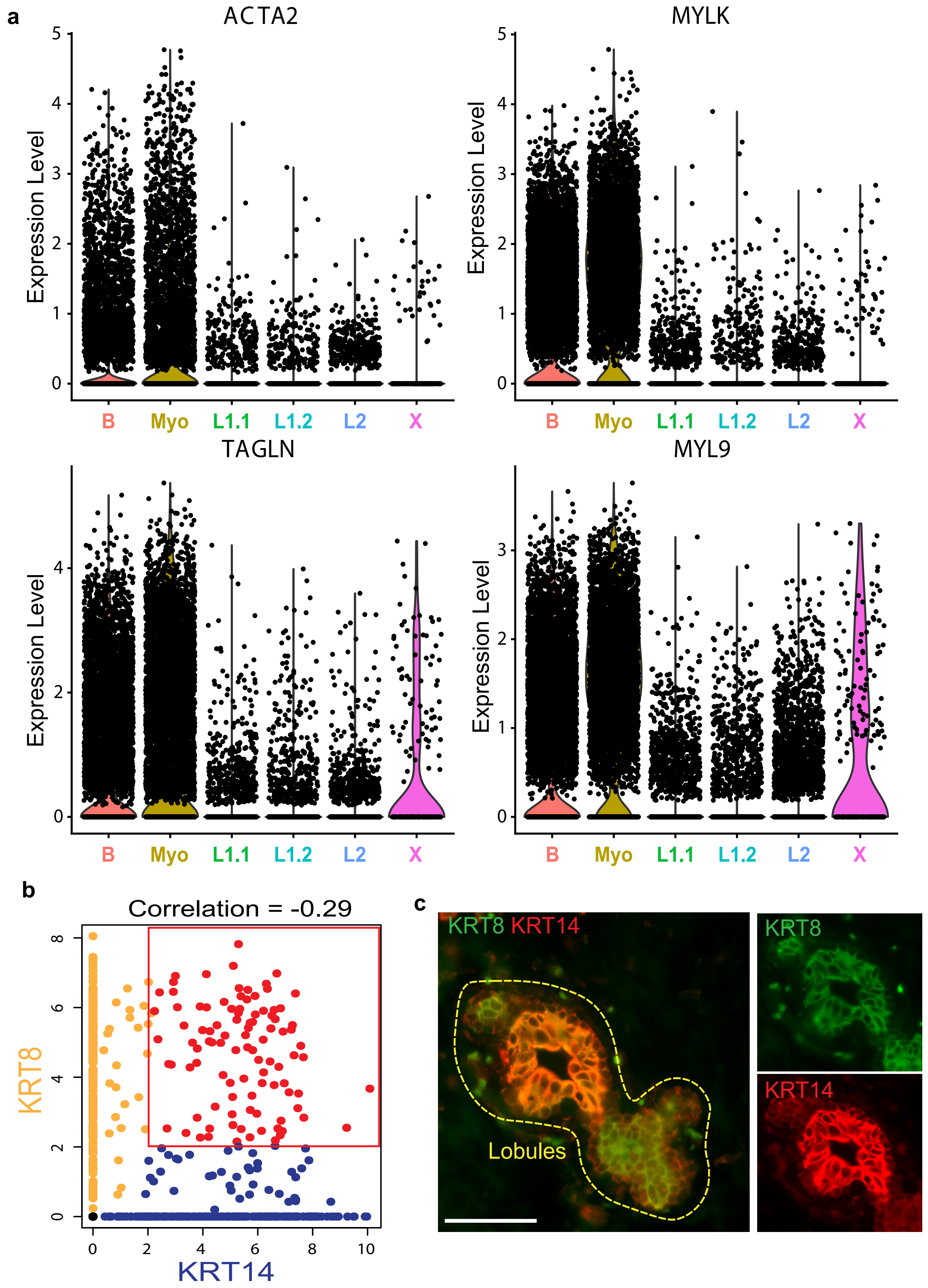

Supplementary Figure 4. Expanded characterization of cellular heterogeneity within the basal compartment. (a) Violin plots of the expression of ACTA2, MYLK, TAGLN, and MYL9 in the combined analyses of the droplet-enabled scRNAseq data grouped by final cluster determination. **(b)** Correlated expression analysis of luminal marker KRT8 and basal marker KRT14 from scRNAseq data revealed a significant number of double positive cells. **(c)** Combined immunostaining for KRT8 and KRT14 showing rare foci of double positive cells in the luminal cell layer of lobular regions. Scale bar = 50 μm.

Supplementary Figure 5

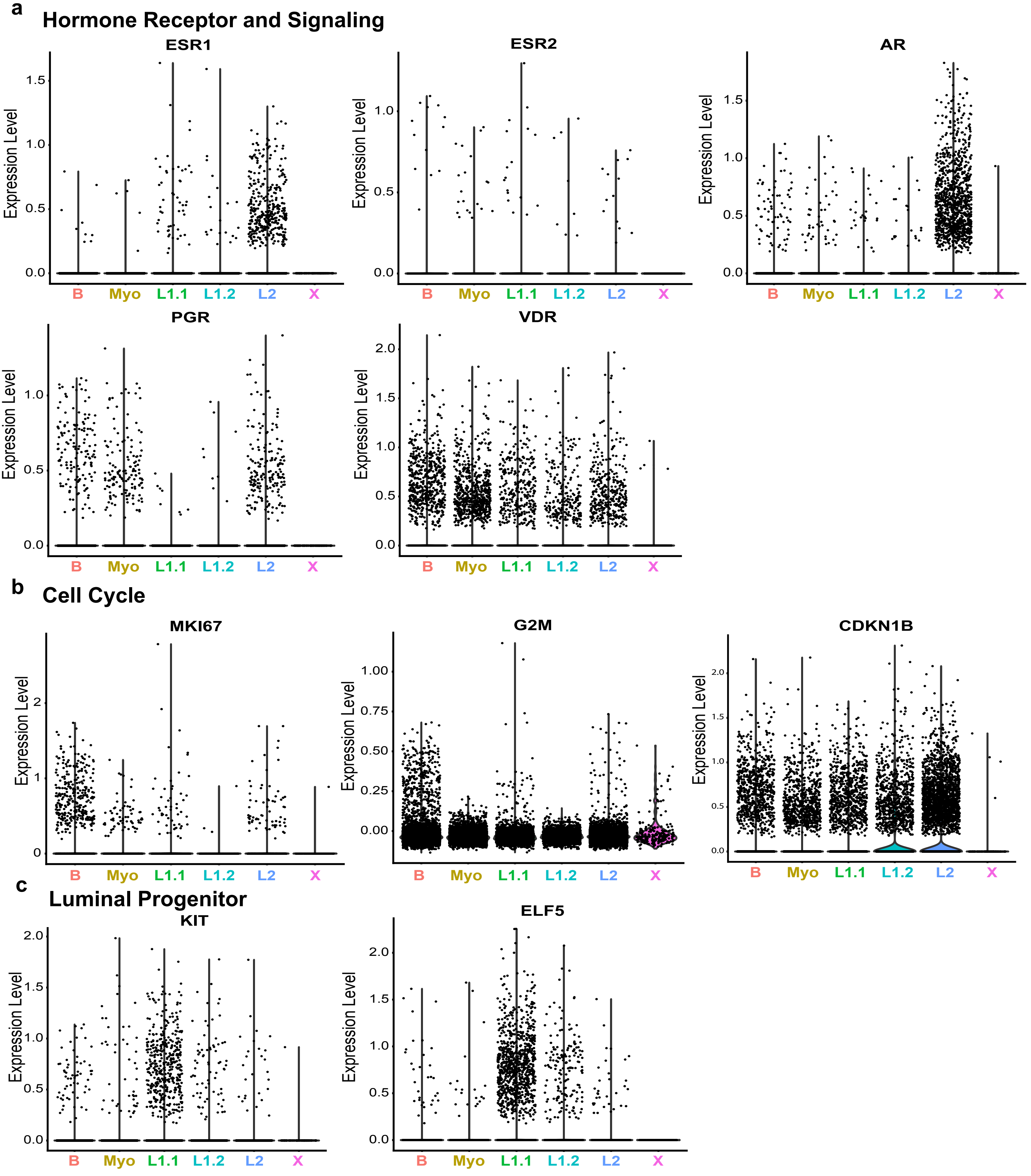

Supplementary Figure 5. Expression patterns for selected genes of interest in combined analysis of droplet-enabled scRNAseq datasets. (a) Violin plots illustrating the expression patterns of hormone receptors, cell cycle genes MKI67 and CDKN1B (p27) and gene scoring for G2M gene signature **(b)**, and luminal progenitor genes **(c)** grouped by final cluster determination.

Supplementary Figure 6

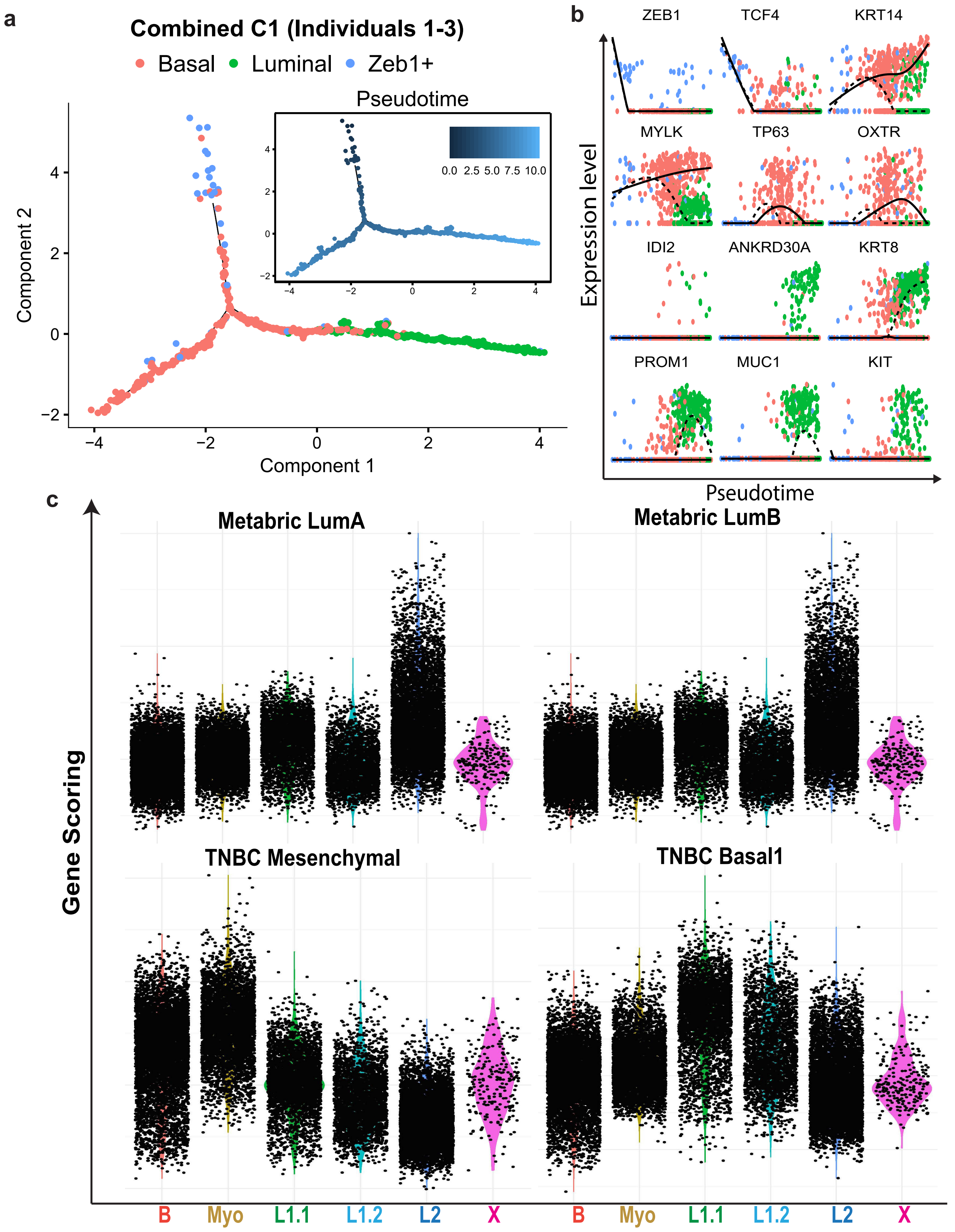

Supplementary Figure 6. Reconstructing breast epithelial lineage hierarchies their relation to breast cancer. (a) Pseudotemporal analysis of microfluidics-enabled scRNAseq results using Monocle2 based on a set of 183 Seurat identified marker genes suggest a differentiation trajectory from ZEB1+ progenitor cells (green) bifurcating into basal (red) and luminal (blue) differentiated cells. **(b)** Selected marker genes are shown as dot plots displayed as expression level over pseudotime. **(c)** Relation of cell states identified in droplet-enabled scRNAseq analysis to different breast cancer subtypes is shown as violin plots displaying gene scoring results for a cells on gene lists derived from breast cancer subtypes, namely Metabric Luminal A (LumA), Metabric Luminal B (LumB), triple-negative breast cancer (TNBC) mesenchymal-like, and TNBC-Basal1.
