# Supplementary material for: Profiling human breast epithelial cells using single cell RNA sequencing identifies cell diversity
Source: Nat Commun. 2018 May 23;9:2028. doi: 10.1038/s41467-018-04334-1 (PMC5966421; doi:10.1038/s41467-018-04334-1)
Supplement: Supplementary file 3 — Description of Additional Supplementary Files [file 41467_2018_4334_MOESM3_ESM.pdf]

Description of Additional Supplementary Files

File Name: Supplementary Data 1
Description: Reduction mammoplasty patients information

File Name: Supplementary Data 2
Description: Analysed genes list
